# Handed Foraging Behavior in Scale-Eating Cichlid Fish: Its Potential Role in Shaping Morphological Asymmetry

**DOI:** 10.1371/journal.pone.0044670

**Published:** 2012-09-06

**Authors:** Hyuk Je Lee, Henrik Kusche, Axel Meyer

**Affiliations:** 1 Lehrstuhl für Zoologie und Evolutionsbiologie, Department of Biology, University of Konstanz, Konstanz, Germany; 2 Zukunftskolleg, University of Konstanz, Konstanz, Germany; 3 International Max Planck Research School for Organismal Biology, University of Konstanz, Konstanz, Germany; 4 Graduate School in Chemical Biology, University of Konstanz, Konstanz, Germany; University Zürich, Switzerland

## Abstract

Scale-eating cichlid fish, *Perissodus microlepis*, from Lake Tanganyika display handed (lateralized) foraging behavior, where an asymmetric ‘left’ mouth morph preferentially feeds on the scales of the right side of its victim fish and a ‘right’ morph bites the scales of the left side. This species has therefore become a textbook example of the astonishing degree of ecological specialization and negative frequency-dependent selection. We investigated the strength of handedness of foraging behavior as well as its interaction with morphological mouth laterality in *P. microlepis*. In wild-caught adult fish we found that mouth laterality is, as expected, a strong predictor of their preferred attack orientation. Also laboratory-reared juvenile fish exhibited a strong laterality in behavioral preference to feed on scales, even at an early age, although the initial level of mouth asymmetry appeared to be small. This suggests that pronounced mouth asymmetry is not a prerequisite for handed foraging behavior in juvenile scale-eating cichlid fish and might suggest that behavioral preference to attack a particular side of the prey plays a role in facilitating morphological asymmetry of this species.

## Introduction

Within-species behavioral polymorphisms are ubiquitous. One particularly interesting case is handed (lateralized) behavior, where individuals exhibit a behavioral bias towards either one or the other side. The most familiar example of lateralized behavior comes from humans, where most individuals (89%) are right-handed and a minority are left-handed (8%) or ambidextrous (3%) [1]. Behavioral lateralization can also frequently be observed in other species; for example, hand-use preference in Chimpanzees [2] and in some birds like Australian parrots [3], and foraging preference in the Japanese snail-eating snakes [4], [5]. This lateralized behavior, particularly in mammals and birds, is thought to be linked to lateralized brain functions and neuro-anatomical asymmetries (reviewed in [6]). From an ecological perspective, handed behavior is suggested to have evolved because it might provide organisms with a selective advantage (e.g. in terms of foraging efficiency in the snail-eating snake, *Pareas iwasakii* [4]; escape performance from predator attacks in the shiner perch, *Cymatogaster aggregata* [7]; predation success in the scale-eating cichlid, *Perissodus microlepis* [8]).

Handed behavior has also been frequently reported in fish, e.g., with respect to eye usage preference (i.e. visual lateralization) in a poeciliid fish [9], [10], swimming-turns in zebra-and goldfish [11] and foraging in a freshwater goby [12] and in some African cichlid fishes [13], [14]. Lateralized behavior in fish is often correlated with morphological asymmetries. In the herbivorous cichlid *Telmatochromis temporalis*, for example, the right mouth morph uses the right side of the jaw more frequently and the left morph the left side [13]. A significant correlation between lateralization in swimming and the anatomical bias of the prevalence of different muscle types was found in zebrafish [11]. However, relatively little effort has been directed towards the exploration of the potential role of handed behavior in facilitating morphological laterality [15].

**Figure 1:**
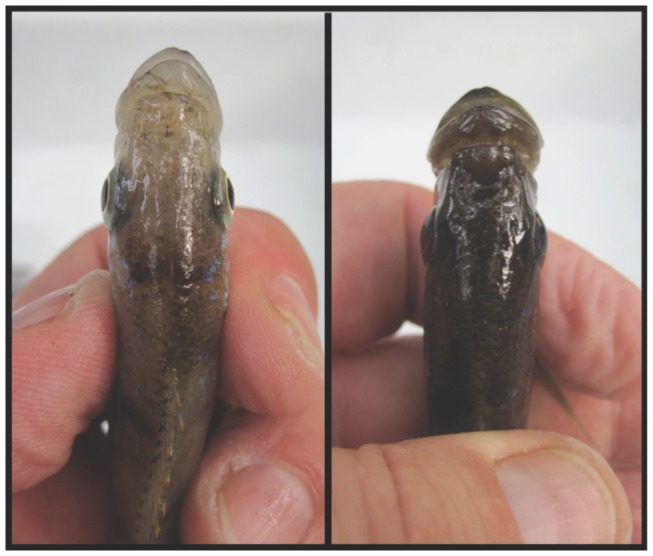
Dorsal view of right-bending (left) and left-bending (right) mouth morphs of the Lake Tanganyikan scale-eating cichlid fish, *Perissodus microlepis*.

A well-known textbook example of a significant interaction between handed behavior and morphological laterality in fish is *Perissodus microlepis*, a scale-eating (lepidophagous) cichlid fish species from Lake Tanganyika [16]. This species is extremely ecologically specialized since most individuals either have a mouth that is bent to the left (‘L-morph’) or to the right (‘R-morph’) (see Figure 1), although a recent study suggests frequent occurrences of fish with a rather symmetrical mouth (Kusche, Lee, Meyer, in revision). L-morphs preferentially attack the right flanks of their prey fish while R-morphs attack the left flanks [8], [17]–[19]. This ‘lateralized (handed) foraging behavior’ therefore represents an extreme form of specialization on a predominantly scale diet that is even restricted to scales from the left or the ride side of prey fish. Yet, previous studies on lateralized scale-eating behavior of *P. microlepis* focused on adult fish only and the presence and strength of this lateralized behavior in juvenile fish have never been tested before. Therefore, how and when these behavioral preferences arise ontogenetically is unknown.

The frequency of L and R morphs in natural populations of *P. microlepis* is suggested to be maintained by negative frequency-dependent selection [8]. Over time, the proportion of L and R morphs within populations oscillates around a 50∶50 ratio [8], [17]. The resulting lateralized foraging behavior of *P. microlepis* is expected to make prey fish more alert to being attacked from the preferred side of the more abundant morph. Thus, increased prey vigilance would reduce the predation success of the more abundant morph, and negative frequency-dependent selection would thereby favor the rarer morph in each generation. Consequently, the frequency of both morphs is maintained in approximately equal abundances [8].

Several important questions about this fish, including the bases of its behavioral and morphological laterality remain unanswered [20]. Mouth laterality of *P. microlepis* has been suggested to be genetically determined by a single Mendelian locus with two alleles: the ‘R’-allele was suggested to be dominant over the ‘L’-allele and ‘R’ was suggested to be homozygous lethal [8], [14], [21]. But a recent review [20] noted that the data reported so far (of mouth-morph ratios in the offspring of parents of known laterality) actually are inconsistent with a single locus Mendelian model. This model was further questioned because the distribution of mouth asymmetry was found to be unimodal rather than bimodal [18], [21] (Kusche, Lee, Meyer, in revision). Phenotypic plasticity may therefore play a role in shaping mouth asymmetry [18] and hence, head asymmetry may be governed by both genetic and environmental factors [18] (Lee *et al*., unpublished data). The genetic and/or environmental basis of behavioral handedness in *P. microlepis*, however, remains largely unexplored.

Furthermore, whether foraging handedness is expressed earlier during development and induces and thereby facilitates mouth asymmetry via phenotypic plasticity [15], [18] or the reverse – remains unclear. Hori [8] originally suggested that mouth laterality in *P. microlepis* is a functional ‘prerequisite’ for efficient lepidophagy. He further proposed that mouth asymmetry (controlled by a single Mendelian locus) precedes and invokes and directs lateralized foraging behavior through natural selection. But, several lepidophagous cichlid species in Lake Tanganyika lack a pronounced laterality in their heads [21] and behavioral preferences have not been tested in these species. Moreover, handed foraging behavior might actually precede, and even induce mouth asymmetry, given the purported role of phenotypic plasticity in mouth laterality [18].

In this study, we examine the strength and individual variation of lateralized behavior and its interaction with mouth laterality in *Perissodus microlepis*. In semi-natural conditions, we conducted feeding experiments on adult wild-caught scale-eaters with their natural prey to test whether pronounced morphological laterality predicts foraging preferences. We further tested whether laboratory-reared juvenile scale-eaters, which had never encountered prey fish before, displayed lateralized scale-feeding behavior in reference to mouth asymmetry. Here we demonstrate relatively strong handedness in foraging behavior in juvenile fish that showed much less mouth asymmetry (compared to wild-caught adult fish) and we then discuss the potential role of lateralized foraging behavior in shaping the head asymmetry of this species.

## Materials and Methods

### Sample collection

Fifty-four breeding pairs of *Perissodus microlepis* were collected in April 2010 by diving with hand nets at Toby Veal's Lodge (S08°37.4′ E031°12′) near Mpulungu (Zambia) on the southern tip of Lake Tanganyika to assess the mating pattern (Kusche, Lee, Meyer, in revision). Twenty-one out of these 54 pairs were used for foraging experiments on adult *P. microlepis* under semi-natural conditions. Mouth laterality of each of the pairs was judged by eye in the field by two independent researchers (H.K. and A.M.) (see Figure 1).

Five broods from different parents of determined mouth-laterality (3 RL and 2 RR pairs) were transported to the animal care facility at the University of Konstanz. In total, 65 young were raised brood-wise in separate 40 *l* and later 200 *l* aquaria with *Artemia* nauplii and flake food. These fish were used for laboratory feeding experiments on juveniles as well as for quantitative measures of mouth asymmetry (Kusche, Lee, Meyer, in revision).

Sixty-one more juveniles from three different broods (3 young of RL, 6 of LL and 52 of RR parental-pairs) were obtained by breeding wild-caught fish in the laboratory. Lab-reared *P. microlepis* fish reached sexual maturity at about six to nine months of age. These 61 juveniles, the F_1_ fish of wild-caught stock, were used for foraging experiments that examined behavioral handedness and its interaction with mouth asymmetry.

Field research was conducted under the study permit (G.R. No: 2077761) granted by the government of the Republic of Zambia (Immigration Department, Ministry of Home Affairs, Republic of Zambia) according to their Immigration and Deportation Act. CAP123, Section 16. Animal care of the fish and all foraging experiments in the laboratory were approved by the regional board of animal welfare in Germany (Regierungspräsidium Freiburg, Abteilung Landwirtschaft, Ländlicher Raum, Veterinär- und Lebensmittelwesen) (permit number: 35/9185.81/G-10/96).

### Field foraging experiments of adult fish

Thirteen outdoor pools (1000 *l*) at the shore of Lake Tanganyika were stocked with one breeding pair of *P. microlepis* each (7 RL, 5 RR and 1 LL pairs). Also two large community tanks of 4000 *l* volume (with 6 L-morphs and 10 R-morphs of *P*. *microlepis*, respectively) were used in these foraging experiments. The cichlid species *Tropheus moorii* (pair tanks: *n* = 3–6; community tanks: *n* = 18 and 25) was used as prey since it is a preferred natural prey species of *P. microlepis* [22]. After 72 hours, all *T. moorii* were removed from the pools and presence/absence of scars and missing scales and the numbers of bites on both flanks of the prey fish were recorded.

Percentages of pooled attacked left (and right) flanks of prey fish were calculated for each tank. We considered these estimates as foraging preference for a particular side, given that the scale-eaters had in principle an equal opportunity to attack both flanks. Foraging preference was further assessed by taking into account foraging scores reflecting different levels of injury (i.e. the amount of damage done by the scale-eaters; 0 bites  = 0 scores; 1–3 bites  = 1 score; 4–6 bites  = 2 scores; >6 bites  = 3 scores). The proportion of these scores for the prey fishes' pooled left (and right) flanks was calculated for each tank.

Two-tailed Fisher's exact probability tests were performed to examine whether in both community tanks the left or right side of the prey fish were preferentially attacked and whether the morphs differed in their foraging scores. A Mann-Whitney-test was performed to test for differences in ratios of affected left flanks of the prey as well as in the amount of foraging scores between RL and RR pairs.

### Laboratory foraging experiments of juvenile fish

Juvenile *P. microlepis* of about two [*n* = 61; mean standard length (SL)  = 3.2 cm; SD = 0.28 cm], three [*n* = 47; total length (TL) = 3–4 cm] and seven [*n* = 24; mean total length (TL) = 7.7 cm; SD = 0.58 cm] months of age, that had not had an opportunity to eat scales from prey fish before, were tested for lateralized foraging behavior. The older test cohorts (that were tested at three and seven months) were caught as 1-2 week old fry in the field, whereas the fish tested at two months were bred in the laboratory (the F_1_ fish of the older cohorts; see above). Eleven of the three-month old fish were re-tested at seven months, but those individuals could not be traced due to logistical reasons. The scale-eaters were placed individually with a single prey fish (platy fish, *Xiphophorus maculatus*, for three-month old fish and goldfish, *Carassius auratus auratus*, for two- and seven-month old fish) in the trial tanks.

Two different methods were used to analyze foraging behavior. For the three-month old fish, one prey fish was added to a 40 *l* aquarium and after 12 hours, the prey fish was examined for scars and missing scales by two different researchers (H.L. and H.K.). This procedure was replicated (2–5 times) for each individual scale-eater to investigate whether its foraging behavior was consistent across a series of 2–5 experimental trials during a period of 1–2 weeks. Because it was impossible to enumerate number of scars and missing scales on the prey fish (*X. maculatus*), foraging preference was assessed for each scale-eater based on observed presence/absence of scars and missing scales. A foraging score of +1 was given for fish that attacked only the right side of prey fish in a particular trial, a score of 0 meant that both sides were attacked, and a score of −1 was given for fish that attacked only the left side. The trials where no scars and no missing scales were observed on the prey fish, or where the prey fish died during the experiments were excluded from the analysis. Since the estimated foraging score of each individual was found to be constant over the trials (e.g. 2 trials: Wilcoxon-signed-ranks-test, *n* = 17, *z* = −0.333, *p* = 1.0; 3 trials: Friedman-test, *n* = 12, Chi-square statistic  = 4.333, *p* = 0.189), the mean foraging score was calculated and used in further analyses. Note that the foraging score of 34 scale-eaters was calculated from the 2–5 trials, while that of 10 individuals was obtained from a single trial only. Only three of the 47 scale-eaters (6%) tested at three months never fed on scales.

To more precisely quantify ‘behavioral’ foraging preference in juvenile *P. microlepis*, a second series of experiments for the seven- and later two-month old scale-eaters was carried out. For each seven-month old individual, its foraging behavior was monitored (in 3–4 replicates during 1–2 weeks) by counting the number of attacks to the left and/or right flanks on a single goldfish, until a total of maximally 20 attacks per individual within up to 30 minutes were reached. The scale-eaters showed reported natural foraging behavior, i.e., they attacked prey from behind [8]. In only a few cases they attacked from the front, but those attacks were not counted. Behavioral foraging preference (i.e. probability of left attack) was again found to be consistent among the 3–4 trials (repeated-measure ANOVA; *F*_3, 45_ = 0.363, *p* = 0.78) as observed in the three-month old fish. Therefore, the handedness scores (e.g. number of left and right attacks) were pooled over the trials to calculate behavioral foraging preference for each scale-eater. The total number of attacks observed per fish ranged from 39 to 80 (mean  = 64). For the two-month old scale-eaters, we employed the same procedure as for the seven-month old fish, except that we conducted only one experimental trial per individual. The average number of attacks observed per fish in this test cohort was 19.

To statistically analyze if juvenile *P. microlepis* fish show a bimodal or unimodal distribution in their foraging behavior (e.g. foraging score, behavioral foraging preference), the dip statistic [23] and a mixture analysis with a parametric bootstrap test (1000 iterations) using the mixtools package [24] were performed in R [25]. An Anscombe-Glynn test [26] for platykurtosis was further performed for the seven-month old fish only (see below).

To investigate whether behavioral foraging preference is translated into foraging score (e.g. number of scales bitten by the scale-eaters), surface areas of attacked left and right flanks of prey (i.e. surface areas of scars and missing scales) were calculated for a sub-sample (*n* = 15) of the two-month old fish. Because individual fish that exclusively attacked one side of the prey only left scars/missing scales at that flank (100%), the 15 fish were selected from individuals that did not forage exclusively from one side (e.g. 0.1< probability of left attack <0.9). The attacked areas of the prey fish were estimated in ImageJ 1.45r (http://imagej.nih.gov/ij) from standardized photographs in a lateral view with an implemented scale. A ratio of the attacked areas (left to right flanks) on the prey fish was calculated for each scale-eater and linear regression analysis was then conducted using probability of left attack as an independent variable (predictor) and the estimated ratio as a dependent (response) variable.

### Relationship between mouth laterality and behavioral handedness in juveniles

The mouth bending angle, ‘α L − β R’ in ° following [14] was measured to test for a relationship between mouth/head asymmetry and handed foraging behavior in juvenile fish. For this test, each live test fish was photographed from a dorsal view in a standardized upright position using a Zeiss Axiophot digital microscope (Zeiss, Germany). The mouth bending angles were then measured in ImageJ 1.45r: on each image, a triangle connecting the most anterior points of the eye sockets and the tip of the snout was drawn to estimate angles (°),α L (angle of the vertex by the left eye) and β R (angle of the vertex by the right eye) (Kusche, Lee, Meyer, in revision).

To evaluate the accuracy of the measurements, repeatability of α L − β R was estimated from repeated and blind measurements that were done from two replicate photographs of the same individuals from sub-samples (*n* = 20, 15 and 15 for the two-, three- and seven-month old fish, respectively). Repeatability, referred to as the proportion of the total variation that is due to variation among individuals, was calculated from one-way ANOVA (individual  =  factor) following [27].

Correlation analyses were performed between mouth bending angles and foraging score (for our test cohort of the three-month old fish) and behavioral foraging preference (probability of left attack for the two- and seven-month old fish) to test for the significant relationship between mouth asymmetry and behavioral handedness. Linear regression analyses were also carried out to test whether mouth asymmetry amplifies as body size increases in the two- and seven-month old fish. For those analyses, SL (standard length) and TL (total length) were used as size measures for the two- and seven-month old fish, respectively.

## Results

### Mouth laterality predicts preferred attack side in adult fish

**Figure 2:**
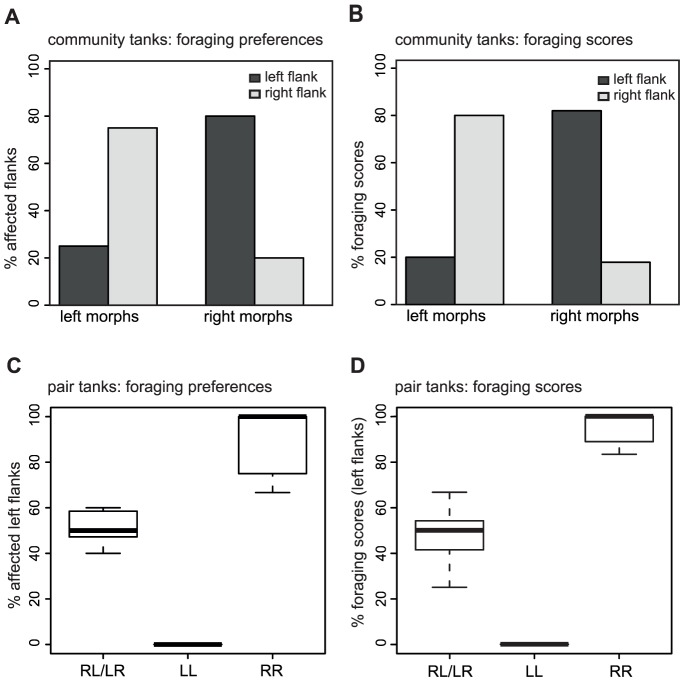
Lateralized foraging behavior in adult *P. microlepis*. Mouth asymmetry strongly predicts foraging preferences and foraging scores in community tanks (A and B) and pair tanks (C and D) of different laterality combinations.

Mouth laterality strongly predicted the preferred attack side as well as foraging scores on either side of the prey fish (Figure 2; Table S1). Both L- and R-mouth morphs from the community tanks clearly exhibited opposed foraging preferences (Fisher's exact probability test: *n* = 41; *p*<0.001) and yielded more scars/missing scales in foraging from their preferred flanks (Fisher's exact probability test: *n* = 63; *p*<0.0001) (Figure 2 A, B). R-morphs preferentially attacked left flanks of the prey fish (80% of affected flanks; 82% of foraging scores). L-morphs preferred to feed from right flanks (75% of affected flanks; 80% of foraging scores).

The same clear pattern was found in the 13 pools with one pair of *P. microlepis* each as predators (Figure 2 C, D): seven RL pairs fed from both flanks with similar frequencies (ratio of attacked left flanks: 40–60%; average: 52%; median: 50%) and produced similar amount of damage onto both flanks (range of foraging scores on left flanks: 25–67%; average: 48%; median: 50%). Five RR pairs strongly preferred to feed from the left flank (range: 67–100%; average: 88%; median: 100%), which caused more bites on that flank (range: 83–100%; average: 94%; median: 100%). A single LL pair exclusively fed from the right flanks of their prey fish. Differences in foraging patterns such as foraging preference and foraging score among morph pair combinations were both highly statistically significant (Wilcoxon-rank-sum-test with continuity correction: proportion of left flanks affected: *w* = 35, *p*<0.01; proportion of foraging scores at the left flank: *w* = 35, *p*<0.01).

### Strong handed foraging behavior in juvenile fish

**Figure 3:**
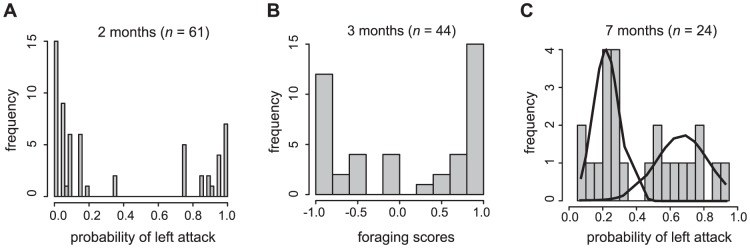
Lateralized foraging behavior in juvenile *P. microlepis*. Frequency distribution of behavioral foraging preference (for two- and seven-month old fish) and foraging score (for three-month old fish) shows a bimodal distribution. (A) two month; (B) three month; (C) seven month old fish. In (C), the graphical inspection of the mixture analysis (fitting two single-component normal distributions to the data) is shown, indicating that the distribution better fits to bimodality than to unimodality, despite a marginal statistical significance of one single-component normal distribution (*p* = 0.057).

Feeding experiments with laboratory-raised juveniles showed that nearly all test fish preyed immediately on scales. Scale-eating behavior is already expressed at an early ontogenetic stage (two-month old: 100%; three-month old: 94%; seven-month old: 100%). Most individuals showed a clear bias to attack only a particular side of their prey and the frequency distribution of the foraging score and behavioral foraging preference clearly exhibited a bimodal distribution (except in the seven-month old fish) (Figure 3). Foraging behavior of the younger test cohorts (of two and three months of age) showed a significant departure from a unimodal distribution (two-month old: dip statistic  = 0.114, *p*<0.001; three-month old: dip statistic  = 0.136, *p*<0.001), whereas the oldest cohort of seven months of age did not (dip statistic  = 0.057, *p*>0.5). The mixture analyses with the parametric bootstrap tests further showed that two-component normal distributions best fitted foraging behavior data of the two- (*p*<0.001) and three-month old fish (*p*<0.001), while one-component normal distribution statistically best fitted the data of the seven-month old fish with marginal significance (*p* = 0.057). However, the graphical inspection of the mixture analysis (Figure 3 C) and a marginal significance of platykurtosis (*p* = 0.092) rather support a weak bimodal distribution [28].

There was considerable ‘inter-individual’ variation in the intensity (strength) of lateralized foraging behavior (Figure 3). Many fish strongly preferred or even exclusively attacked the left or the right sides [e.g. 7 of 61 (12%) and 15 of 61 (25%) of the two-month old fish foraged exclusively from the left and the right sides, respectively], while other fish displayed a less pronounced bias in foraging behavior (Figure 3).

**Figure 4:**
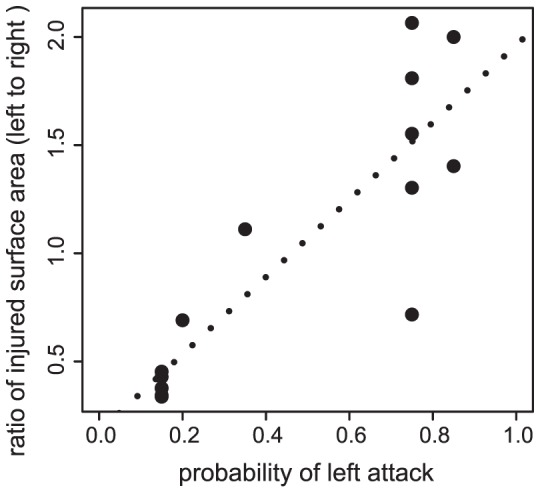
Relationship between behavioral foraging preference and foraging score. Lateralized foraging behavior and foraging score (e.g. number of scales eaten by the scale-eaters) are highly significantly correlated (in a sub-sample [*n* = 15] of the two-month old fish [*y* = 1.787*x*+0.176, *R^2^* = 0.759, *p*<0.001]).

As predicted, a highly significant positive correlation was found between behavioral foraging preference and foraging score in the subset (*n* = 15) of the two-month old juveniles (*y* = 1.787*x*+0.176, *R^2^* = 0.759, *p*<0.001; Figure 4), suggesting that foraging score is an outcome of behavioral attack preference. This result further indicates that our field data on foraging preference and foraging score of wild-caught adult fish could indeed reflect ‘behavioral’ foraging preference.

### Lack of correlation between handed behavior and mouth asymmetry in juveniles

Our measurements of mouth bending angles appeared to be fairly repeatable: estimated repeatability of the mouth bending angles was 0.80, 0.77 and 0.87 for the two-, three- and seven-month old fish, respectively. Those estimates of the repeatability imply that 77 to 87% of the total observed variation is attributed to underlying ‘true’ variation in the mouth bending angles among individuals and the remaining 13 to 23% variation is due to measurement error.

**Figure 5:**
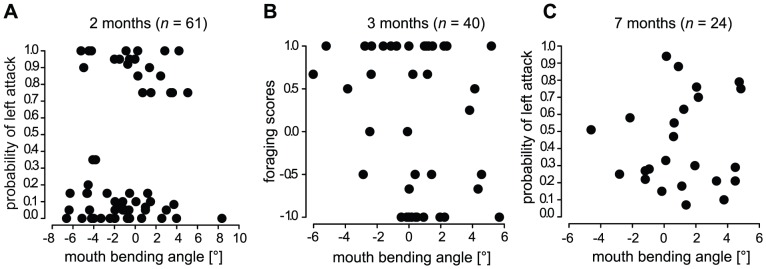
Relationship between mouth asymmetry and lateralized foraging behavior. Mouth asymmetry (mouth bending angle) is not significantly correlated with foraging handedness in juvenile *P. microlepis*. (A) two month (*r* = 0.148; *p* = 0.255); (B) three month (*r* = −0.229; *p* = 0.154); (C) seven month old fish (*r* = 0.069; *p* = 0.749). Note that 11 fish were tested at two different ontogenetic stages (i.e. at three and seven months of age).

Neither of the juvenile cohorts showed a significant correlation between mouth asymmetry and lateralized foraging behavior (two-month old fish: *r* = 0.148; *p* = 0.255; three-month: *r* = −0.229; *p* = 0.154; seven-month: *r* = 0.069; *p* = 0.749; Figure 5). Unexpectedly, some fish that were morphologically scored as (slightly) R-morphs (with negative values of mouth bending angle of α L − β R) occasionally even attacked the right side more frequently than the left side, and vice versa (Figure 5). This lack of correspondence suggests that mouth laterality is not a prerequisite for handed foraging behavior for juvenile *P. microlepis*. Also, the level of mouth asymmetry (i.e. absolute values of mouth bending angles) of the laboratory-reared juvenile fish did not significantly increase with body size in either two- (*n* = 61, *y* = 0.831*x*+0.04, *R^2^* = 0.015, *p* = 0.35) or seven- (*n* = 24, *y* = −0.496*x*+5.394, *R^2^* = 0.032, *p* = 0.4) month old fish.

## Discussion

The handedness of the foraging behavior and the associated asymmetry in mouth/head morphology have made the scale-eating cichlid fish, *Perissodus microlepis*, a textbook example [16] of both, the astonishing degree of ecological specialization and negative frequency-dependent selection [8]. However, how and when lateralized foraging behavior manifests itself during ontogeny, and whether its association with mouth asymmetry is already apparent in juvenile individual fish had remained untested. Here, we report on the strength and individual variation of lateralized foraging behavior as well as its relationship with mouth asymmetry in *P. microlepis* during its juvenile as well as adult life stages. We find that handed foraging behavior is already prominent at an early age (e.g. at two-months), although the initial morphological asymmetry is less evident, which hints that handed behavior might play a role in bringing about pronounced morphological laterality, considering the potential influences of phenotypic plasticity on mouth asymmetry [18].

The observed strong lateralization in foraging behavior in young scale-eaters (e.g. bimodal distribution) that was not accompanied by notable morphological asymmetry (see also Kusche, Lee, Meyer, in revision) and the obvious correspondence between mouth orientation and foraging behavior in adult fish might suggest that handed behavior is probably expressed earlier during development. And, it may actually induce and facilitate morphological asymmetry [18], if phenotypic plasticity plays a relatively larger role than the genetic determination of this trait (Lee *et al*., unpublished data). An alternative hypothesis is that both handed behavior and morphological laterality are genetically governed, but expressed at different ontogenetic stages. However, this hypothesis would seem to be rather unlikely given our observation that laboratory-reared fish of now about two-year of age still have a relatively symmetrical mouth (HL, personal observation).

The field foraging experiments with adult fish clearly demonstrate that manifested mouth laterality corresponds to a pronounced bias in feeding laterality. This finding is consistent with previous studies that discovered strong correlations between mouth laterality and handed foraging behavior in adult fish [8], [17]–[19]. However, not every adult individual exclusively attacked the prey flank according to its scored mouth laterality (see Figure 2; Table S1 in the current study and [17]). The observed level of individual variation in the degree of lateralized foraging behavior might translate into varying expression of mouth asymmetry (see Kusche, Lee, Meyer, in revision), provided – and this may be a strong assumption – that mouth laterality, but not foraging preference are appreciably influenced by environmental factors [18]. The observed consistency of foraging behavior in the three- and seven-month old juveniles over the repeated experiments during 1–2 weeks supports the hypothesis that handed behavior is not particularly plastic (over that time-scale), which is consistent with the previous study [18]. How much of the observed morphological differences are initially brought about by a (heritable?) behavioral bias that, through phenotypic plasticity becomes also fixed on a morphological level remains to be tested.

However, we observed a rather more pronounced laterality in foraging behavior among the younger juvenile fish (e.g. at two and three months), compared to the fish at seven months. The observed dwindling laterality in foraging behavior in the older fish might imply that feeding preference is expressed at an early age (e.g. at two months), but the initial level of laterality would diminish over time (under laboratory conditions) unless the fish were constantly to feed on scales. Yet, whether this trend means the strength of handed behavior truly decreases with age awaits future experiments on “tracked” individuals over a series of ontogenetic stages during their life time.

The foraging experiments with juvenile fish in the laboratory show that mouth asymmetry does not predict handedness in foraging behavior, possibly due to the small degree of mouth asymmetry. Surprisingly, young and still quite small scale-eaters preyed on scales of prey fish and exhibited pronounced handed behavior (Figure 3). Even the two-month old fish readily fed on scales of similar- or even slightly larger-sized goldfish. However, the degree of mouth asymmetry in juvenile scale-eaters was rather small [on average only 2.67° (for two-month: SD = 1.92°), 2.01° (three-month: SD = 1.73°) and 2.12° (seven-month: SD = 1.61°)] and the relationship between behavioral bias and morphological asymmetry was always non-significant (Figure 5). Note that the degree of mouth asymmetry in those laboratory-reared scale-eaters is indeed substantially lower than in wild-caught adult *P. microlepis* (the average  = 5.07°; *n* = 238; SD = 3.51°; Kusche, Lee, Meyer, in revision). This too supports the hypothesis that handed behavior might play a significant role in shaping the asymmetry of mouths in *P. microlepis*.

Nonetheless, the observed lack of correlation between mouth asymmetry and lateralized foraging behavior in juvenile *P. microlepis* might also result from the measurement technique used for the quantification of mouth asymmetry (i.e. mouth bending angle) not fully capturing the existing true laterality (i.e. asymmetric skeletal features) in the mouth/head apparatus of this fish. Further tests with cleared and double-stained juvenile fish samples of known behavioral laterality are required to check this possibility.

We here argue that the hypothesis – ‘handed behavior preceding and driving mouth asymmetry’ [15], [18] – seems more strongly supported by evidence than the original hypothesis [8] that mouth asymmetry precedes and directs lateralized foraging behavior through natural selection acting on a single gene. Different lines of evidence support this hypothesis. In a parallel study, we observed a large amount of variation in mouth asymmetry in 238 wild-caught adult specimens and found a continuous and unimodal (and not bimodal) trait distribution (Kusche, Lee, Meyer, in revision). If plasticity rather than genetics plays a comparatively larger role (Lee *et al*., unpublished data), then this unimodal distribution of laterality might simply be the outcome of different levels of lateralization in foraging behavior. This hypothesis is supported by the findings from the foraging experiments of juvenile fish: juvenile fish did not show complete lateralization of foraging preference and even some individuals attacked equally often at both flanks. Whether symmetrically attacking fish might have a potential selective advantage over left or right preferentially attacking fish needs to be tested.

We have indirect evidence that phenotypic plasticity has an influence on mouth asymmetry. The observed relatively symmetrical mouth morphology of the laboratory-reared fish even at the age of seven months (the degree of mouth angles is similar to the two- and three-month old juveniles; see above), would lend support to the hypothesis that phenotypic plasticity considerably contributes to the mouth laterality [18], because the fish did not have prior opportunities to feed on scales, but were fed almost exclusively on regular flake food. It seems that mouth asymmetry could not manifest itself under the laboratory condition with regular food (HL, personal observation). The lack of positive association between the level of mouth asymmetry and body size in the laboratory-reared juvenile fish further supports this hypothesis. By comparison, in wild-caught adult fish mouth asymmetry tends to increase with size (Kusche, Lee, Meyer, in revision), which would be expected if mouth asymmetry were to amplify over an individual's lifetime as a phenotypically plastic response to repeated attacks from one particular side [15], [18].

A significant role of phenotypic plasticity in the evolutionary origin of novel morphologies has been suggested repeatedly during the last several decades [15], [29]–[32]. Phenotypic plasticity clearly contributes to shaping the morphology of the jaw and the mouth apparatus in teleost fishes, particularly in cichlids [33], [34]. Even different food types or diet hardness can induce changes in the external shape of the head during the ontogeny of some cichlids [33], [35]. The teleost skeleton can quickly adapt to changing external factors, so called ‘mechanical adaptation’, and skeletal phenotypic plasticity in teleosts seems to be rather pronounced and taxonomically widespread [36].

Through phenotypic effects of “use and disuse”, handed behavior has been shown to drive morphological laterality in different animal groups (e.g. lobsters [37]; snakes [4], [5]; humans [38]) (reviewed in [15]). Lobsters provide a clear example of how claw asymmetry is shaped during development as a function of handed behavior [37]. Laboratory experiments demonstrated that differential use of claws during early juvenile stage induces and facilitates development of a crusher claw [37]. As such, in *P. microlepis* lateralized behavior might conceivably lead to an asymmetric remodeling of the structural elements (e.g. bones) involved in defining mouth shape [39], given that lateralized behavior in fish sometimes has a strong additive genetic component [40], [41], e.g., the estimated heritability of laterality of eye preference in the poeciliid fish, *Girardinus falcatus* is 0.5 to 0.6) [40]. Although we are uncertain whether handed scale-eating behavior is genetically programmed (innate), rather than environmentally plastic (learning) or both [42], the bimodal trait distribution in very young fish (Figure 3) speaks for a major genetic locus determining handedness in scale-eating behavior ([18]).

In a broader context, our study provides information on a long-standing controversy over the role of behavior in facilitating developmental morphological changes in an adaptive direction (e.g. phenotypic accommodation [43], [44]) and in subsequent evolution of novel forms (e.g. [15], [45]). If behavior-induced morphological innovations provide an organism with improved performance in a given environment, such a behavioral response may secure an individual's survival and reproduction and thus direct available variation in the following generation if traits involved in this behavioral response or the responsiveness *per se* are heritable [46]. Handed behavior-induced mouth laterality in *P. microlepis* is believed to be functionally significant and selectively advantageous (in regard to feeding success) [19]. This line of thinking is reminiscent of C.H. Waddington's ideas about “genetic assimilation” [47], an idea that is in line with recent findings in epigenetics and might merit renewed attention and research effort.

## Supporting Information

Table S1 Lateralized foraging behavior in adult *Perissodus microlepis*: foraging preferences and foraging scores.
(DOC)
