# Supplementary material for: Handed Foraging Behavior in Scale-Eating Cichlid Fish: Its Potential Role in Shaping Morphological Asymmetry
Source: PLoS One. 2012 Sep 6;7(9):e44670. doi: 10.1371/journal.pone.0044670 (PMC3435272; doi:10.1371/journal.pone.0044670)
Supplement: Table S1 — Lateralized foraging behavior in adult Perissodus microlepis: foraging preferences and foraging scores. (DOC) [file pone.0044670.s001.doc]

**Table S1. Lateralized foraging behaviour in adult *Perissodus microlepis*: foraging preferences and foraging scores.**

|  | experimental tank | number of  prey fish | left flanks affected | right flanks affected | foraging scores left flank | foraging scores right flank |
| --- | --- | --- | --- | --- | --- | --- |
| community | left (6 x L) | 18 | 4 | 12 | 5 | 20 |
|  | right (10 x R) | 25 | 20 | 5 | 31 | 7 |
| pair tanks | disassortative 1 (RL) | 5 | 4 | 5 | 6 | 9 |
|  | disassortative 2 (RL) | 3 | 3 | 2 | 6 | 6 |
|  | disassortative 3 (RL) | 3 | 1 | 1 | 1 | 1 |
|  | disassortative 4 (RL) | 3 | 2 | 2 | 3 | 4 |
|  | disassortative 5 (RL) | 3 | 3 | 2 | 6 | 3 |
|  | disassortative 6 (RL) | 3 | 2 | 3 | 2 | 6 |
|  | disassortative 7 (RL) | 4 | 4 | 3 | 7 | 5 |
|  | assortative left 1 (LL) | 6 | 0 | 3 | 0 | 4 |
|  | assortative right 1 (RR) | 3 | 3 | 1 | 8 | 1 |
|  | assortative right 2 (RR) | 3 | 2 | 1 | 5 | 1 |
|  | assortative right 3 (RR) | 3 | 1 | 0 | 1 | 0 |
|  | assortative right 4 (RR) | 5 | 5 | 0 | 12 | 0 |
|  | assortative right 5 (RR) | 3 | 2 | 0 | 6 | 0 |
